# Dystrophic muscle distribution in late-stage muscular dystrophy

**DOI:** 10.4322/acr.2020.221

**Published:** 2020-11-20

**Authors:** Celeste Michelle Pilato, Melissa Sue Walker, Andrea M. Nguyen, McKay Elizabeth Hanna, Scott Lanxing Huang, Erika Morgan Lutins, M. Alex Meredith, Peter Jacob Haar, Mathula Thangarajh, Hope Theresa Richard, Woon Nam Chow

**Affiliations:** 1 Virginia Commonwealth University, School of Medicine, Richmond, VA, USA; 2 Virginia Commonwealth University, School of Medicine, Department of Anatomy and Neurobiology; 3 Virginia Commonwealth University, School of Medicine, Department of Radiology; 4 Virginia Commonwealth University, School of Medicine, Department of Neurology; 5 Virginia Commonwealth University, School of Medicine, Department of Pathology

**Keywords:** Muscular Dystrophies, Musculoskeletal System, Cadaver, Muscular Dystrophy, Limb-Girdle

## Abstract

There is scant information about the comprehensive distribution of dystrophic muscles in muscular dystrophy. Despite different clinical presentations of muscular dystrophy, a recent multi-center study concluded that phenotypic distribution of dystrophic muscles is independent of clinical phenotype and suggested that there is a common pattern of involved muscles. To evaluate this possibility, the present case report used cadaveric dissection to determine the whole-body distribution of fat-infiltrated, dystrophic muscles from a 72-year-old white male cadaver with adult-onset, late-stage muscular dystrophy. Severely dystrophic muscles occupied the pectoral, gluteal and pelvic regions, as well as the arm, thigh and posterior leg. In contrast, muscles of the head, neck, hands and feet largely appeared unaffected. Histopathology and a CT-scan supported these observations. This pattern of dystrophic muscles generally conformed with that described in the multi-center study, and provides prognostic insight for patients and the physicians treating them.

## INTRODUCTION

Muscular dystrophy (MD) has a general occurrence of ~20 per 100,000 people^1^ and its genetic basis has received considerable investigative effort. Correlations between specific gene mutations and different phenotypic expressions are also well established^2^ and result in a range of disorders. Each disorder varies in severity, age of onset and apparent pattern of affected muscles.^3^ The disease affects muscles through selective muscle fiber necrosis and replacement by fat and connective tissue.^4^ However, a survey of the literature reveals scant detail about the phenotypic distribution of affected muscles. Toward that end, a recent magnetic resonance imaging (MRI) study examined the distribution of dystrophic muscle in patients with genetically confirmed dysferlinopathy.^5^ That investigation revealed that muscles of the posterior leg and thigh, and the rectus femoris muscle were most commonly affected, as were trunk, pelvic and periscapular muscle groups. Another study^6^ used MRIs of the lower extremity to reveal a similar pattern of muscle degeneration in the leg and thigh. Curiously, the reported patterns of dystrophic musculature were similar regardless of their specific clinical presentation.^5^ The assertion that the phenotypic distribution of involved muscles is independent of clinical phenotype, however, is based on the resolution of the MRI technique, which identifies individual large muscles. In contrast, the present case study utilizes the precise resolution of cadaveric dissection to isolate and examine all the muscles of an end-stage muscular dystrophy patient to evaluate whether this comprehensive sample of muscles is consistent with the pattern outlined by the MRI study.^5^ Furthermore, because the muscle biopsy is useful when an observed phenotype fails to correlate with that predicted by molecular/genomic analysis,^7^ it is important to know which muscles are most likely to be affected by the disease process as well as those which might not.

## CASE REPORT

A 72-year-old white male (approximately 95.2 kg, 1.82 m in height, BMI 28.5) cadaver was donated for anatomic study. Due to the anonymity of cadaver donation, clinical history was unavailable. Cause of death was attributed to sepsis secondary to a grade 3 sacral bedsore with contributing causes of heart and renal failure. However, during initial dissection in our medical anatomy course, muscles in the shoulders and hips were found to be fat-replaced and a form of muscular dystrophy was suspected. From donor information and public records, it was learned that the donor had been in military service and developed an unspecified form of muscular dystrophy in his adult life. In an attempt to determine the specific genotype of MD in the donor, DNA was submitted for a comprehensive MD panel (Invitae, San Francisco CA), but was of insufficient quality to provide results.

According to U.S. National Institutes of Health guidelines,^8^ the present protocols do not require Institutional Review Board review or approval, given the nature of this study on a cadaveric (non-living) subject. Use of this cadaver for the present study conforms with the ethical principles of the World Medical Association’s Declaration of Helsinki, the guidelines of the Virginia Commonwealth University School of Medicine as well as the donor-signed agreement with the University of West Virginia Human Gift Registry “[...] to donate remains for anatomical study in the advancement of scientific and medical education and research.”

**Figure 1:**
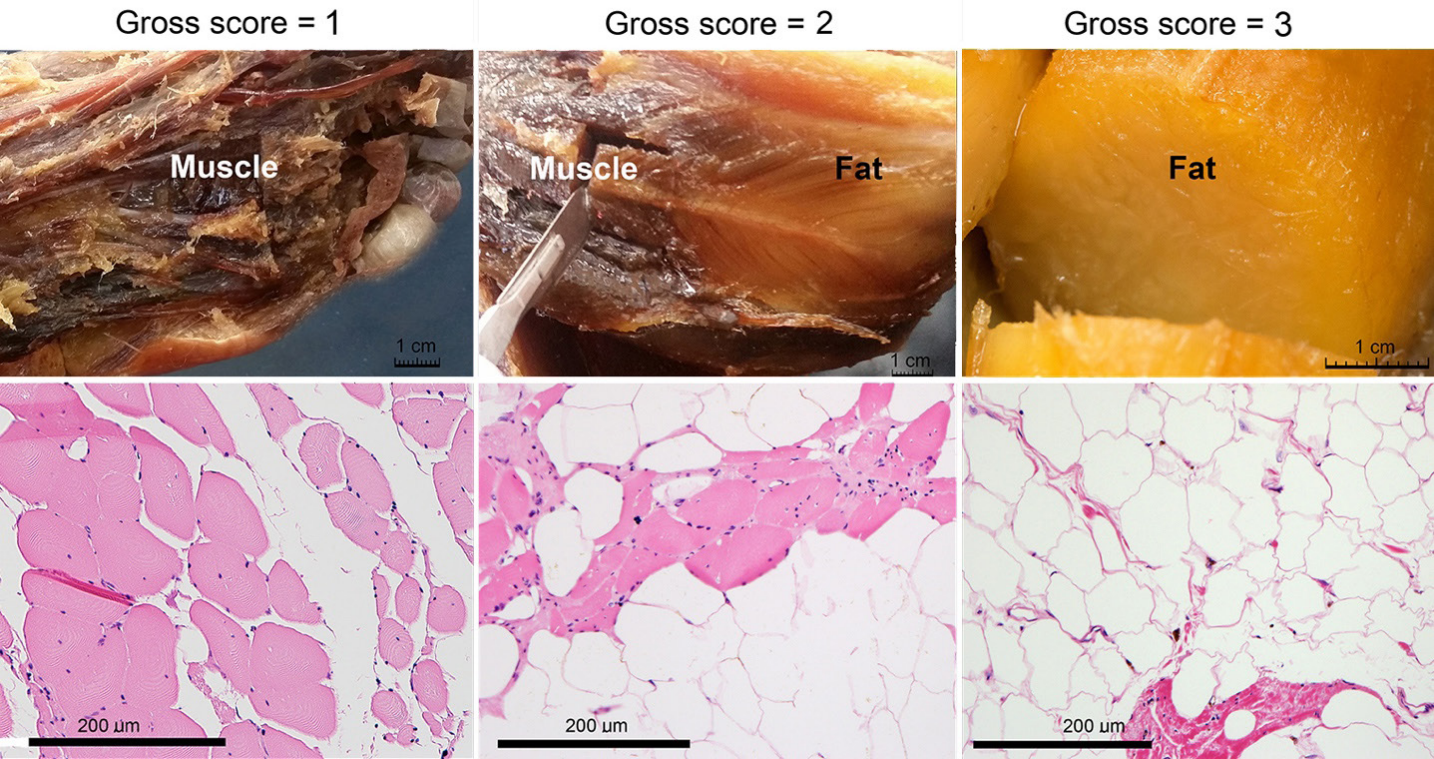
Gross and histological appearance of selected muscles from case study of a 72-year-old white male with late-stage, adult onset muscular dystrophy. A representative gross image and its fat replacement score are shown (top-right) for a muscle with total fat replacement (score=3): the Tensor fascia lata; for a muscle with partial fat replacement (middle; score=2): the Soleus; and for a muscle with no apparent fat replacement (top left; score=1): the Flexor hallucis brevis. Examples of each of these levels of fat replacement are also illustrated at the histological level (bottom row) where photomicrographs of sections through the Pectoralis major muscle (right), Soleus (middle), and Flexor hallucis brevis (left) are shown. Stain: Hematoxylin and Eosin.

To directly evaluate the phenotypic distribution of dystrophic muscles in this case, each skeletal muscle was identified by dissection, examined and assigned one of three scores based on its gross appearance: 1-no grossly-detectable fat replacement, 2-partial fat replacement, and 3-complete fat replacement. Complete fat replacement was apparent as the presence of yellow fat within the fascial envelope that had contained the muscle and lacked muscle fiber remnants. Partial fat replacement was indicated when muscle fibers were interspersed with yellow fat, or the muscle body exhibited a lighter color and spongier texture than normal. Another form of partial fat replacement occurred where intact muscle fascicles were interrupted by zones of complete fat replacement. Differences in distribution of these muscle categories were tested statistically using analysis of variance (ANOVA; p<0.05). Examples of gross observations and scoring categories are depicted in Figure 1 (top) and the gross findings for all examined muscles are tabulated in Table 1.

**Table 1:** This tabulates the grossly observed fat-replacement scores for each muscle exposed in a case study of a 72-year-old white male cadaver with adult-onset, late-stage muscular dystrophy. Scores (as defined in the text) represent no apparent fat infiltration into the muscle (1), a mixture of fat and muscle fascicles (2), or total fat replacement (3). For comparison, also listed are percentage fat replacement values (“NI” = Not Infiltrated; “*” measured as a muscle group) for selected muscles calculated from MRI scans from.^5^

UPPER EXTREMITY	Score	MRI (%)		LOWER EXTREMITY	Score	MRI (%)		AXIAL	Score	MRI (%)
SHOULDER GIRDLE				PELVIC GIRDLE				TORSO/TRUNK		
Trapezius	1			Psoas major/minor	2			Iliocostalis	2	93
Latissimus dorsi	2	75		Iliacus	3			Longissimus	2	86
Rhomboid major/minor	2	70		Quadratus lumborum	2			Spinalis	1	
Levator scapulae	2			Tensor fascia latae	3	95		Transversospinals	1	89
Deltoid	2			Gluteus maximus	3			Serratus posterior sup/inf.	1	
Teres major	2			Gluteus medius	3			External intercostal	1	
Teres minor	2			Gluteus minimus	2	91		Internal intercostal	1	
Supraspinatus	2	73		Piriformis	3			Innermost intercostal	1	
Infraspinatus	2	74		Obturator ext/int.	3	86		Transversus thoracis	1	
Subscapularis	2	81		Gemellus sup/inf.	3			Subcostalis	1	
Pectoralis major	3			Quadratus femoris	3			Levator costarum	1	
Pectoralis minor	3							Diaphragm	1	
PROXIMAL (ARM)								Rectus abdominis	1	
Triceps brachii long/lat/dp.	2	57		PROXIMAL (THIGH)				External abdominal oblique	2	
Coracobrachialis	2			Sartorius	2			Internal abdominal oblique	2	
Biceps brachii long/short	2	57		Rectus femoris	2			Transversus abdominis	1	
Brachialis	2			Vastus lateralis	2			Cremaster	1	
				Vastus intermedius	2			NECK		
				Vastus medialis	3			Longus capitis	1	
				Biceps femoris long/short	2	94		Scalene ant/mid/post.	1	
				Semitendinosus	2	90		Sternocleidomastoid	1	NI
				Semimembranosus	2	95		Splenius	1	25*
				Gracilis	2			Semispinalis capitis	1	25*
				Pectineus	2			Platysma	1	
				Adductor brevis	2			Cricothyroid	1	
				Adductor longus	2	94		Arytenoidius	1	
				Adductor magnus	2	94		Thyroarytenoid	1	
DISTAL (FOREARM)				DISTAL (LEG)				Cricoarytenoid post/lat.	1	
Extensor digitorum	1			Tibialis anterior	2			Digastric	1	
Extensor digiti minimi	1			Extensor hallucis longus	1			Stylohyoid	1	
Extensor carpi ulnaris	1			Extensor digitorum longus	1			Mylohyoid	1	
Extensor carpi radialis long.	2			Gastrocnemius	2	99		Geniohyoid	1	
Extensor carpi radialis brev.	2			Soleus	2	99		Sternohyoid	1	
Brachioradialis	2			Popliteus	1			Sternothyroid	1	
Supinator	2			Flexor hallucis longus	1			Thyrohyoid	1	
Extensor indicis	1			Flexor digitorum longus	1			Omohyoid	1	
Extensor pollicis long/brev.	1			Tibialis posterior	1			Longus colli	1	
Abductor pollicis longus	1			Fibularis longus	2			HEAD / FACE		
Pronator teres	2	54*		Fibularis brevis	1			Occipitalis	2	
Flexor carpi ulnaris	2	54*						Frontalis	2	
Flexor carpi radialis	2	54*						Orbicularis oculi	2	
Flexor digitorum superf.	2	54*						Procerus	1	
Flexor digitorum profund.	2	54*						Levator labii superioris	1	
Flexor pollicis longus	1	54*						Levator anguli oris	1	
Palmaris longus	1							Depressor anguli oris	1	
Pronator quadtratus	1							Depressor labii inferioris	1	
HAND				FOOT				Orbicularis oris	1	
Flexor pollicis brevis	1			Extensor digitorum brevis	1			Buccinator	1	
Abductor pollicis brevis	1			Extensor hallucis brevis	1			Risorius	1	
Opponens pollicis	1			Abductor hallucis	1			Zygomaticus major/minor	1	
Adductor pollicis	1			Abductor digiti minimi	1			Superior rectus	1	
Flexor digiti minimi	1			Flexor digitorum brevis	1			Medial rectus	1	
Abductor digiti minimi	1			Quadratus plantae	1			Lateral rectus	1	
Opponens digiti minimi	1			Lumbricals	1			Inferior rectus	1	
Palmaris brevis	1			Flexor hallucis brevis	1			Superior oblique	1	
Lumbricals	1			Adductor hallucis	1			Inferior oblique	1	
Dorsal interossei	1			Flexor digiti minimi brevis	1			Masseter	1	NI
Palmar interossei	1			Dorsal interossei	1			Temporalis	1	NI
				Plantar interossei	1			Medial pterygoid	1	
								Lateral pterygoid	1	
								Genioglossus	1	34*
								Hyoglossus	1	34*
								Styloglossus	1	34*
										

A head-to-foot summary of gross findings is as follows. Gross changes in the musculature of the head, neck, oral and extraocular muscles were not observed. Muscles of the back largely showed partial, patchy fat infiltrate noticeable among the muscle fibers; these included the Latissimus dorsi, Rhomboids, Levator scapulae, and Erector spinae group (but not the Transversospinal group). Muscles of the anterior chest wall were unaffected (Intercostals, Transversus thoracis) as were some of the anterior abdominal wall (Rectus abdominis, Transversus abdominis) while External and Internal abdominal oblique muscles showed partial fat infiltration. Muscles of the proximal upper extremity were strongly affected, with the Pectoralis major/minor group showing complete fat transformation while all the others were partially affected. All muscles of the upper arm were partially fat-infiltrated while mixed effects between partial and unaffected were observed in the forearm musculature. In contrast, none of the intrinsic muscles of the hand showed gross dystrophic changes. The muscles of the lower extremity seemed more susceptible, with many of them completely replaced by fat. Such completely dystrophic muscles included the Gluteus, Obturator and Gemellus groups, the Iliacus, Quadratus femoris and the Tensor fascia lata. Muscles of the thigh appeared less profoundly affected, but all showed fat infiltration among intact muscle fascicles. In the leg, only the Gastrocnemius, Soleus and Fibularis longus showed partial fat infiltration, with the remainder appearing to be unaffected, as were all the intrinsic muscles of the foot.

**Figure 2:**
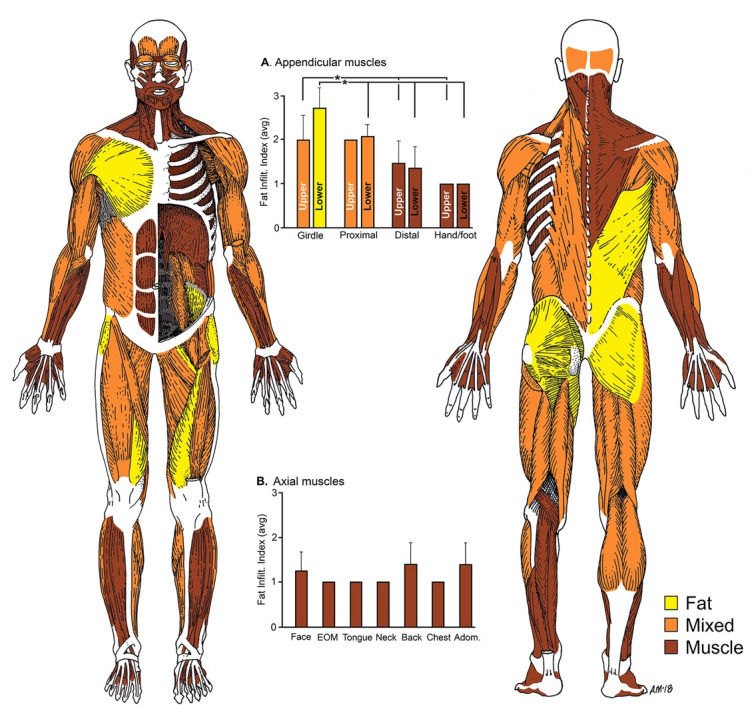
Summary of dystrophic muscle distribution in a case of a 72-year-old white male with late-stage, adult-onset muscular dystrophy. The human musculature is depicted from anterior (left) and posterior (right) views showing both superficial and deep muscle planes. Colors indicate level of fat infiltration as grossly observed during dissection where yellow = complete fat replacement (score=3); orange = mixture of fat and muscle fascicles (score=2); brown = no apparent fat infiltrate in the muscle (score=1). Each muscle was identified and given a score and tabulated by region. These values are graphed where, in part (A) the average (± standard deviation) scores for appendicular muscles were significantly (“*”; p<0.001, ANOVA) higher among limb girdle muscles (pelvis, shoulder) than in the other regions, while those for axial muscles, shown in part (B) appeared to be uniformly unaffected.

**Figure 3:**
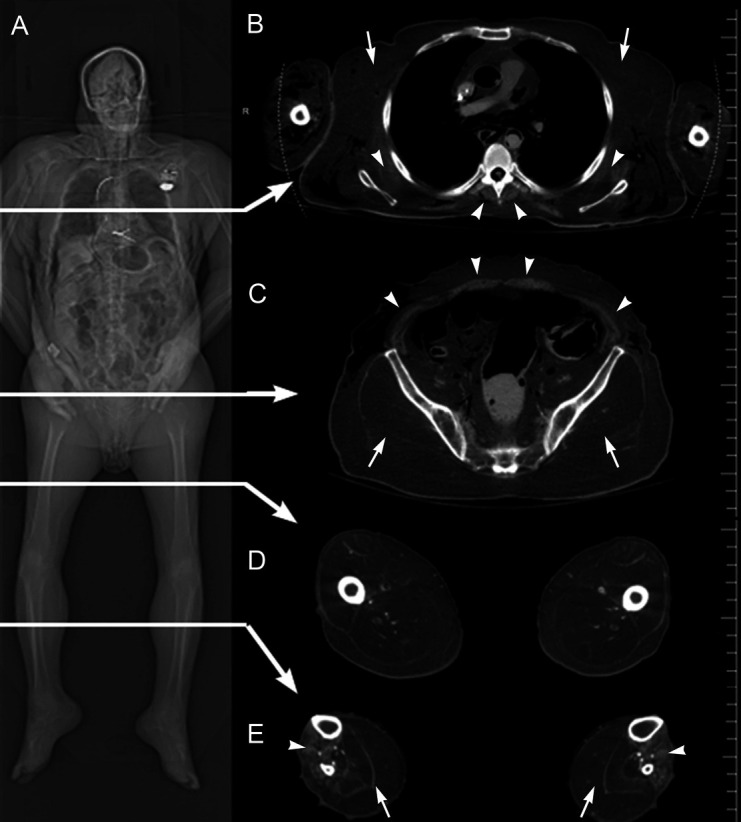
CT images from a case study of a 72-year-old white male with late-stage, adult onset muscular dystrophy. Part (A) is a frontal topogram of the entire cadaver showing diffuse fatty infiltration of skeletal muscles. Arrows indicate levels from which axial sections (B-E) are taken. In (B), the axial CT image is from mid-chest level, showing marked fat replacement of the pectoral muscles (arrows), and partial sparing of the serratus anterior and paraspinal muscles (arrowheads). Soft tissue gas in the upper arms and posterolateral back are related to the embalming process. In (C), at the level of the mid-pelvis, there is striking fatty replacement of the gluteal muscles (arrows), and partial sparing of the musculature of the abdominal wall (arrowheads). In the axial image in (D) at mid-thigh level, there is significant fatty replacement of the musculature of the anterior, posterior, and medial compartments of the thigh. The axial CT image in (E) is at the level of the mid-calf, which shows fatty replacement of the musculature of the leg, with substantial replacement and pseudohypertrophy of the posterior compartment musculature (arrows), and partial sparing of the anterior compartment musculature (arrowheads).

These observations are summarized graphically by the whole-body muscle depiction in Figure 2. Pseudohypertophy of the Gastrocnemius muscles was observed, as is apparent from the CT-scan shown in Figure 3.

Also observed in dissection: the heart was large, but did not meet criteria for cardiomegaly, and both a pacemaker and a defibrillator were found. The diaphragm appeared normal/uninfiltrated and lungs appeared normal. The spleen was small (6 cm x 3 cm). The kidneys also seemed small for body size (right 9 cm long; left 10.5 cm long), but revealed a normal cortex and pelvic structure. This cadaver had thin-walled and dilated large intestines and megacolon, but all other gastrointestinal organs (pancreas, stomach, duodenum, liver) appeared normal. Five cholesterol gallstones (~1 cm^3^) were found in the gallbladder. The central nervous system (brain, spinal cord) was also grossly examined and judged normal. The peripheral nervous system was found grossly intact with no signs of thinning or atrophy in the brachial or lumbosacral plexii.

Statistical analyses (graphed in Figure 2) indicates that proximal muscles were significantly (ANOVA, p<0.001) more affected than distal muscles in both upper and lower extremity, while axial muscles were largely unaffected. When sorted for laterality, little difference was observed between sides (ANOVA, not significant), indicating symmetric bilateral involvement and only 4 muscles showed categorically more fat infiltrate on the left side than the right: Flexor carpi ulnaris, Flexor digitorum profundus, Adductor brevis, Adductor magnus. Conversely, only the Extensor carpi radialis showed more fat infiltrate on the right side than the left. Anterior-posterior biases in dystrophic muscle distribution were also compared, but were not found to be statistically significant.

Biopsies were collected from selected skeletal muscles (n=19) and visceral organs (n=12) for standard histological assessment. Post-mortem biopsy specimens were formalin-fixed, paraffin-embedded, sectioned at 4 µm and counterstained with hematoxylin and eosin. Photomicrographic images were collected using a Nikon Eclipse E600 microscope with an Olympus DP72 camera. Within each image, the percentage of fat replacement was quantified by measuring relative cross-sectional areas (fat to fascicle area) using the Image processing program; multiple images of each sample were evaluated to determine a mean value. Representative histopathology samples are depicted in Figure 1 (bottom) and comparison of gross categorization and histologically-determined fat-replacement scores showed a general correspondence among their assessments (R^2^=0.35). The Pectoralis muscles (which had gross score = 3; total fat replacement) showed highest values (>95%) of fat replacement at the microscopic level; Rhomboids, Deltoid and Infraspinatus (gross score=2; partial) revealed 39%, 45% and 41% fat replacement, respectively. Adductor pollicis brevis, Lumbricals and Extensor hallicus brevis (gross score=1; no fat replacement) each showed lowest values (<5%). However, on microscopic analysis, some muscles showed measurable amounts of fat infiltrate when they did not appear to contain fat infiltrate at the gross level (Masseter = 28%; Medial rectus = 13%; Lateral rectus = 11%). A biopsy sample from the diaphragm showed minimal (<5%) fat replacement. Similarly, no fat infiltration was observed histologically in cardiac or viscera biopsies taken at gastrointestinal levels from mouth to anus.

Prior to dissection, a full-body CT scan was obtained in one helical acquisition using a Siemens (Munich, Germany) Somatom Definition Flash CT scanner. Slice thickness was 0.6 mm, kV 140, and mA 280. Axial, sagittal, and coronal images were reconstructed with 5.0 mm slice thickness. The frontal CT image (Figure 3) revealed dispersed replacement of skeletal muscle with fat-density tissue. An axial CT image at the mid-chest level shows profound muscle loss in the region of the pectoral muscles but partial sparing of the serratus anterior and paraspinal muscles. The axial CT image at mid-pelvic level reveals fat replacement of the gluteal muscles with partial sparing of the musculature of the abdominal wall. The axial CT image at mid-thigh level indicates fatty replacement of the musculature in all compartments (anterior, posterior, and medial). Finally, the axial CT image at the mid-calf level depicts the extensive replacement of the posterior compartment musculature but partial sparing of the anterior compartment. Overall, the CT images indicate the selective but pervasive replacement of skeletal muscle with fat-density tissue, most prominently in areas of the proximal appendicular skeleton of the pectoral and gluteal regions.

## DISCUSSION

The present case study provides a muscle-by-muscle analysis of dystrophic muscle distribution from an elderly adult patient with late-stage muscular dystrophy. The most profoundly dystrophic muscles were found in the proximal upper and lower limbs while apparently unaffected muscles were located largely in the head, neck, and distal upper (hands) and lower (feet) extremities. This pattern of dystrophic muscles supports the general description reported for late-stage patients with dysferlinopathy (;^5^, see list in Table 1). In addition, the increased resolution afforded by dissection indicates that the distribution and proportion of affected muscles appears more extensive than revealed with MRI. Furthermore, selected histopathological analysis reveals that subtle levels of dystrophy may be present even in muscles that appear normal at the gross level. On the other hand, the muscles of the hands, feet and visceral systems largely appear to be spared from dystrophic effects.

A limitation of the present case study is that samples were collected from an anonymous post-mortem, formalin-fixed donor. As such, muscle specific diagnostics were attempted but unsuccessful. Furthermore, patient records were not available under the terms of the donation and attempted genetic tests were not successful. Therefore, to appraise the specific type of MD in this case, it is important to consider similarly-presenting muscular dystrophies, such as Becker MD and Duchenne MD. There was no dilated cardiomyopathy noted in this case, which would be an unusual presentation for BMD or DMD because dilation becomes more apparent with age in both of these.^9^ ^,^ ^10^ Neck muscle involvement, which is common in BMD and DMD^11^ is also lacking in this case. Therefore, based on the gross pattern of affected muscles, the histological characteristics with the calculated fat to fascicle percentage, the apparent lack of cardiac involvement, as well as age and size of the patient, a form of adult-onset Limb Girdle Muscular Dystrophy (LGMD) is suspected. There are several commonalities in dystrophic muscle distribution described for dysferlinopathies of which LGMD is a subgroup,^5^ but in this case, other forms of LGMD may also be possible. Ultimately, the present comprehensive assessment of dystrophic muscle distribution in an end-stage, adult MD patient provides valuable insight into the extent of muscle involvement and, as such, offers considerable prognostic value.
